# A novel rice *fragile culm 24* mutant encodes a UDP-glucose epimerase that affects cell wall properties and photosynthesis

**DOI:** 10.1093/jxb/eraa044

**Published:** 2020-02-17

**Authors:** Ran Zhang, Huizhen Hu, Youmei Wang, Zhen Hu, Shuangfeng Ren, Jiaying Li, Boyang He, Yanting Wang, Tao Xia, Peng Chen, Guosheng Xie, Liangcai Peng

**Affiliations:** 1 Biomass & Bioenergy Research Centre, College of Plant Science & Technology, Huazhong Agricultural University, Wuhan, China; 2 State Key Laboratory of Biocatalysis & Enzyme Engineering, College of Life Science, Hubei University, Wuhan, China; 3 College of Life Science & Technology, Huazhong Agricultural University, Wuhan, China; 4 University of Manchester, UK

**Keywords:** Arabinogalactan protein, cellulose biosynthesis, cell wall, fragile culm, galactolipids, photosynthesis, rice, UDP-glucose epimerases

## Abstract

UDP-glucose epimerases (UGEs) are essential enzymes for catalysing the conversion of UDP-glucose (UDP-Glc) into UDP-galactose (UDP-Gal). Although UDP-Gal has been well studied as the substrate for the biosynthesis of carbohydrates, glycolipids, and glycoproteins, much remains unknown about the biological function of UGEs in plants. In this study, we selected a novel rice *fragile culm 24* (*Osfc24*) mutant and identified it as a nonsense mutation of the *FC24/OsUGE2* gene. The *Osfc24* mutant shows a brittleness phenotype with significantly altered cell wall composition and disrupted orientation of the cellulose microfibrils. We found significantly reduced accumulation of arabinogalactan proteins in the cell walls of the mutant, which may consequently affect plant growth and cell wall deposition, and be responsible for the altered cellulose microfibril orientation. The mutant exhibits dwarfism and paler leaves with significantly decreased contents of galactolipids and chlorophyll, resulting in defects in plant photosynthesis. Based on our results, we propose a model for how OsUGE2 participates in two distinct metabolic pathways to co-modulate cellulose biosynthesis and cell wall assembly by dynamically providing UDP-Gal and UDP-Glc substrates.

## Teaser

A novel rice *fragile culm* mutant (*Osfc24*) possesses a nonsense mutation of *OsUGE2* encoding a UDP-Glc epimerase, which participates in metabolic pathways that affect both cell wall properties and photosynthesis.

## Introduction

Plants possess a complex system of families of nucleotide sugar interconversion enzymes for biosynthesis of universal sugar donors (Reiter and Vanzin, 2001; Bar-Peled and O’Neill, 2011). In this system, UDP-glucose epimerase (UGE; EC.5.1.3.2) is required for interconverting UDP-Glc to UDP-Gal. UGEs are essential for the *de novo* synthesis of UDP-Gal, which serves as a building block for the biosynthesis of carbohydrates, glycolipids, and glycoproteins (Barber *et al.*, 2006). UGEs can also take up galactose into catabolic metabolism and hence detoxify plants from galactose stress (Dörmann and Benning, 1998).

Genes encoding UGEs have been characterized in various plant species. To date, the Arabidopsis genome is known to encode five UGE isoforms, with AtUGE2, AtUGE4, and AtUGE5 preferentially catalysing the conversion of UDP-Glc to UDP-Gal, whilst AtUGE1 and AtUGE3 function in the opposite direction (Barber *et al.*, 2006). Genome-wide reverse genetic analysis has demonstrated that AtUGE2 and AtUGE3 influence pollen development, AtUGE2 and AtUGE4 synergistically provide galactose and promote plant growth, and AtUGE1 and AtUGE5 provide functional support for AtUGE4 (Rösti *et al.*, 2007). In addition, AtUGE4 is required for the galactosylation of xyloglucan and type II arabinogalactan (AG), but not for the biosynthesis of galactolipids (Seifert *et al.*, 2002; Barber *et al.*, 2006; Rösti *et al.*, 2007). Four genes encoding UGEs have been identified in rice (Kim *et al.*, 2009). OsUGE1 plays an important role in cell wall carbohydrate partitioning during limiting nitrogen conditions (Guevara *et al.*, 2014), and transgenic OsUGE1-overexpression lines contain more galactose and glucose from non-cellulosic polysaccharide fractions. An unannotated rice PHD1 protein has been shown to possess UGE activity with specific localization in the chloroplast, and has also been shown to be an essential enzyme for the biosynthesis of galactolipids and for photosynthetic efficiency (Li *et al.*, 2011). UGE overexpression in potato increases the galactose content in tuber cell walls (Oomen *et al.*, 2004), and transgenic rice expressing *Brassica rapa* BrUGE1 shows improved plant growth and drought tolerance (Abdula *et al.*, 2016). Although the function of some UGEs in plants has been reported, much remains unknown their roles in cell wall biosynthesis and assembly, as well as in photosynthesis.

Characterization of brittle-culm mutants has proved to be an effective way to study genes involved in cell wall formation and polymer assembly (Zhang and Zhou, 2011). In Arabidopsis, several important genes have been identified for the biosynthesis of wall polymers and the maintenance of the cell cytoskeleton (Turner and Somerville, 1997; Taylor *et al.*, 1999, 2000; Brown *et al.*, 2007; Hu *et al.*, 2018*b*). In rice, about a dozen *brittle culm* (*bc*)/*fragile culm* (*fc*) mutants have been identified with defects in cell wall formation. For instance, *BC1* encodes a COBRA-like protein that is essential for cell wall assembly in mechanical tissues (Li *et al.*, 2003), and *BC3* encodes a classic dynamin-related protein, OsDRP2B, withs function in the biosynthesis of secondary cell wall cellulose (Hirano *et al.*, 2010; Xiong *et al.*, 2010). *BC7*, *BC11*, and *FC16* encode cellulose synthases (CESAs) for cellulose biosynthesis (Yan *et al.* 2007; Zhang *et al.*, 2009; Li *et al.*, 2017). *BC12/GDD1* encodes a kinesin-4 protein that controls the progress of the cell-cycle and that may function in the microtubule-dependent deposition of cellulose (Zhang *et al.*, 2010). *BC15* encodes a chitinase-like protein that belongs to the glycosyl hydrolase (GH) family and that plays an important role in cellulose synthesis (Wu *et al.*, 2012). To date, a number of genes have been characterized in different plant species to participate in various metabolic processes including cell wall biosynthesis, vesicle trafficking, and cytoskeleton conformation. However, it remains to be determined how the supply of active nucleotide sugars regulates cell wall formation and plant photosynthesis, which in turn affect plant growth and development.

In this study, we identified a novel rice *fragile culm 24* mutant (*Osfc24*) encoding UDP-glucose epimerase (designed as OsUGE2) that can interconvert UDP-Glc and UDP-Gal. Because OsUGE2 mainly has enzyme activity in providing UDP-Gal as the substrate for the biosynthesis of AGP and chloroplast galactolipids, we examined how its mutation resulted in a typical culm brittleness phenotype by affecting cell wall deposition and the orientation cellulose microfibril. We also observed stunted plant growth and a paler leaf color due to reduced chlorophyll contents and inhibition of photosynthesis. Based on our results, we propose a model for the involvement of OsUGE2 in distinct metabolic pathways for the dynamic modulation of cellulose biosynthesis and cell wall assembly in rice.

## Materials and methods

### Plant materials and growth conditions

The rice (*Oryza sativa*) *fragile culm 24* (*Osfc24*) mutant was selected from a T-DNA mutagenesis pool of the *japonica* variety Nipponbare in 2008 and was grown to obtain homozygous plants in 2011. An F_2_ mapping population was generated by crossing the *Osfc24* mutant and Minghui 63 (MH63), a wild-type polymorphic *indica* variety. All plants used in this study were cultivated in an experimental field at Huazhong Agricultural University (Wuhan, China).

### Measurement of mechanical properties

At the plant heading stage, the 2nd internodes were chosen to test the mechanical properties. The stretching force was measured using a universal force-length testing device (model RH-K300, Guangzhou Runhu Instruments Co. Ltd., China). The breaking force of the internodes was determined using a Prostrate Tester (DIK 7401, Daiki Rika Kogyo Co. Ltd., Japan) with the same fulcrum each time.

### Map-based cloning and complementary genetic study

To identify the gene of the *Osfc24* mutant an F_2_ mapping population was generated by crossing *Osfc24* and MH63, and a total of 206 mutant plants were screened for mapping with the markers listed in Supplementary Table S1 at *JXB* online. To conduct a genetic complementary study, the entire coding sequence (CDS) region of *OsUGE2* driven by its own promoter was transformed into the pC3300T vector to generate the transformation plasmid *pFC24F*. The full sequence of the complementary fragment (3044 bp) was amplified and fused using Promoter-UGE2 primers and CDS-UGE2 primers. The complementary plasmid was introduced into *Agrobacterium tumefaciens* strain EHA105 and transformed into the *Osfc24* plants as described previously (Lin and Zhang, 2005). CDS-UGE2 primers were also used to confirm that *pFC24F* was transformed into the *Osfc24* mutant. *Mse I-UGE2* primers were used to amplify fragments covering the mutation site of *Osfc24*, then the digestion of *Mse I* on this fragment allowed the generation of polymorphisms between the wild-type (WT) and mutant. All primers used for functional studies of *OsUGE2* are listed in Supplementary Table S2.

### Microscopic observations and quantitative analysis of images

Sections of the 2nd internode of plants at the heading stage were stained with Toluidine Blue and Calcofluor White, and were then imaged using a light microscope (BX-61, Olympus). The lengths and widths of cells (*n*≥50) were measured using the ImageJ 1.32j software (https://imagej.nih.gov/ij/).

TEM was used to observe the thickness of cell walls in the veins of the third leaf in seedlings at the 3-leaf stage, as described previously (Li *et al.*, 2017). Samples were post-fixed in 2% (w/v) OsO_4_ for 1 h after extensively washing in PBS buffer and embedded using a Spurr Low Viscosity Embedding Kit (Sigma). Sections were cut using an Ultracut E ultra-microtome (Leica) and picked up onto formvar-coated copper grids. After post-staining with uranyl acetate and lead citrate, the specimens were viewed under a Hitachi H7500 TEM.

Atomic force microscopy (AFM) was used to observe the structure of cellulose microfibrils. The basal region of the 2nd internodes of plants at the heading stage were sectioned using a microtome (VT1000S, Leica). To remove lignin with a minor effect on cellulose, we used treatment with 8% acidic chlorite (1 mM HCl added to 1 g sodium chlorite) at 50 °C incubation, with two cycles of 24 h each. The sections were then washed with pure water at least five times and stored for subsequent AFM observations. We used a PicoPlus Molecular Imaging system together with a PicoScan 3000 Controller, and an Agilent multipurpose AFM scanner with open-loop was used for imaging. The whole system was situated on a PicoPlus Isolation Chamber to avoid environmental noise. All images were taken using non-contact, top magnetic AC (TopMAC) mode under PicoTREC (Agilent Technologies) and images of the topography were collected. All samples were imaged at an average scanning speed of one line per second with 512×512 pixels. At least three independent images were taken as biological replicates.

### Analysis of subcellular localization

To determine the subcellular localization of OsUGE2, the full-length cDNA sequences were transformed into the vector pCAMBIA1302. Primers are listed in Supplementary Table S2. OsUGE2 was fused with green fluorescent protein (GFP) under the control of the Cauliflower mosaic virus (CaMV) 35S promoter and subsequently transformed into rice protoplasts, which were obtained from the stem tissues of 2-week-old seedlings as described previously (Yoo *et al.*, 2007). Observations of GFP fluorescence were performed using a TCS SP8 confocal laser scanning system (Leica Microsystems).

### Cloning and recombinant expression of OsUGE2

The plasmid pET28a (Stratagene) was used as the expression vector in *E. coli*. The coding region of *OsUGE2* was amplified by PCR from the leaves of WT and *Osfc24* plants using the primers 28a-OsUGE2-F/R (Supplementary Table S2). The PCR product was purified using a gel extraction kit (DH101, Biomed, Beijing), digested with the restriction enzymes *Bam*H I and *Hind* III, and then ligated into the pET28a vector, which was digested with the same enzymes. The recombinant vector was transformed into *E. coli* BL21 (DE3) for protein expression. The positive clones were screened by PCR and restriction enzyme digestion and then sequenced by Sangon Biotech Co., Ltd. (Shanghai, China). The positive clones were then cultured in LB medium containing 50 μg ml^–1^ kanamycin at 37 °C in a shaker with a rotation speed of 220 rpm. When the optical density (OD_600_) of the culture medium reached 0.6, isopropylthiogalactoside (IPTG) was added at a final concentration of 0.5 mM, and the culture was further grown at 18 °C for 18 h.

All purification steps were performed at 4 °C at a flow rate of 1 ml min^–1^ unless otherwise stated. The culture broth (300 ml) was sampled by centrifuging at 8000 *g* for 5 min. The precipitate was collected, resuspended in PBS buffer (pH 7.5), and ultrasonicated. The cell debris was then removed by centrifuging at 8000 *g* for 5 min, and the supernatant was used as the crude extract. His·Bind Kits (Novagen) were then used to purify the His-tagged proteins following the manufacturer’s instructions. The concentrate was loaded onto a GE HiTrap Desalting column equilibrated with 20 mM Tris/HCl (pH 8.0) buffer. The molecular weight and homogeneity of the protein were evaluated by 12% SDS-PAGE.

### Total protein extraction

Second internodes of rice stem tissues at the plant heading stage were ground into powder with liquid nitrogen, and then extracted with 50 mM Tris/HCl buffer (pH 8.6). After centrifugation at 12 000 *g* for 10 min at 10 °C, the supernatants were filtered (10 kD, Millipore) and collected for UGE activity assays.

### UGE enzyme activity assays

UGE enzyme activities were determined as described previously (Kotake *et al.*, 2009) with some minor modifications. Briefly, the reaction mixture containing 50 mM Tris/HCl buffer (pH 8.6), 0.1 mM NAD^+^, 1 mM UDP-sugar, and 10 μg enzyme in a volume of 100 μl. The reaction was conducted at room temperature for 15 min and terminated using a boiling water bath for 10 min. The reaction products were then hydrolysed by adding 10 μl of 1 M HCl for 10 min at 90 °C for 10 min, after which 10 μl of 1 M NaOH was added. The amount of released sugars was analysed by GC-MS. One unit of enzyme activity is defined as the amount of enzyme converting the sugar moiety of 1 μmol of UDP-sugar into its epimer per min (expressed as U mg^–1^). The concentration of protein was determined by the Bradford method of with BSA as the standard.

### Immunolabeling

Samples of the 2nd internode from WT and *Osfc24* plants at the heading stage were cut into 8-μm cross-sections using a paraffin slicer (RM2265, Leica). Immunolabeling procedures were conducted as described previously (Hu *et al.*, 2018*c*, 2019), with some minor modifications. The LM6, JIM8, JIM13, MAC207, and CCRC-M7 antibodies against arabinogalactan (AG) were used (Seifert *et al.*, 2002; Zhou *et al.*, 2009; Qin *et al.*, 2017). The fluorescence intensity in different zones of the images was quantified using the ImageJ software.

### Yariv staining and treatments

The 2nd and 3rd internodes from WT and *Osfc24* plants at the heading stage were used for cross-sectioning. The sections were incubated with β-glucosyl Yariv reagent (β-GlcY, 1 mg ml^–1^) for 1 h at room temperate, washed with distilled water three times, and visualized by microscopy. Germinated rice seedlings were cultured in water with β-glucosyl Yariv reagent (50 μM) for 5 d, and the roots were then collected for RNA extraction and transcription analysis of *OsCESA*s.

### Determination of galactose content in galactolipids

Total lipids were extracted from WT and *Osfc24* plants at 30–90 d old as described previously (Chetal *et al.*, 1983), with minor modifications. Briefly, the chloroplasts were shaken with CHCl_3_–MeOH–H_2_O (5:10:4) and purified with CHCl_3_. The lower CHCl_3_ phase that contained the galactolipids was further hydrolysed with 0.1 M KOH (dissolved in pure alcohol). Distilled water was then used to extract galactose, which was quantified using the anthrone test.

### Determination of chlorophyll content and photosynthetic rate

Pigments were extracted from leaves of 60-d-old plants using 95% ethanol solvents, and were quantified using colorimetric methods as described previously (Sartory and Grobbelaar, 1984). The uppermost fully expanded leaves were chosen for measurements of photosynthetic rate, stomatal conductance, and transpiration rate using a LI-6800 portable photosynthesis system (LI-COR), as described previously (Wang *et al.*, 2018).

### Determination of cellulose properties

Crude cellulose degree of polymerization (DP) assays were performed using the viscosity method as described previously (Zhang *et al.*, 2013). Cellulose crystalline index (CrI) was determined using X-ray diffraction (XRD; Rigaku-D/MAX instruments, Ultima III, Japan) using a standard method (Segal *et al.*, 1959).

### Determination of monosaccharide composition of non-cellulosic polysaccharides by GC-MS

Crude cell-wall residues (40 mesh) were obtained by removing soluble sugars, lipids, and starch. The residues were hydrolysed with 2 M trifluoroacetic acid (TFA) and neutral sugars were determined by GC-MS (Shimadzu GCMS-QP2010 Plus) with *Myo*-inositol as the internal standard, as the described previously (Xu *et al.*, 2012). Three biological replications were performed.

### Analysis of cell wall composition

The procedure for fractionation of the plant cell walls was as described previously (Peng *et al.*, 2000; Jin *et al.*, 2016). Soluble sugars, pectin, hemicelluloses, and cellulose fractions were determined as described previously (Cheng *et al.*, 2019). Colorimetric methods were applied for determination of hexoses, pentoses, and uronic acid as previously described (Huang *et al.*, 2019). Total lignin was assayed using a two-step acid hydrolysis method according to the Laboratory Analytical Procedure of the National Renewable Energy Laboratory (Sluiter *et al.*, 2008).

### RNA isolation and qRT-PCR analysis

RNA extraction and qRT-PCR analysis was conducted as described previously (Hu *et al.*, 2018*a*). Total RNA was isolated from the collected tissues using Trizol reagent (Invitrogen). The first-strand cDNA was obtained using OligodT and M-MLV reverse transcriptase (Promega). qRT-PCR amplification was carried out on a Bio-Rad MyCycler thermal cycler with SYBER Premix ExTaq (Takara) according to the manufacturer’s instruction, and *OsUBQ* was used as the internal control. The PCR thermal cycle conditions were as follows: one cycle of 95 °C for 2 min, followed by 45 cycles of 95 °C for 15 s, 58 °C for 15 s, and 72 °C for 25 s. The expression level of genes was normalized to that of *OsUBQ*, which was set as 100. Primers used in these assays are listed in Supplementary Tables S2, S3. Three biological replications were performed.

### Biomass enzymatic digestibility

To examine biomass enzymatic digestibility, chemical pre-treatments followed by mixed-cellulase hydrolysis were conducted as described previously (Huang *et al.*, 2019). Briefly, cell wall samples were incubated with 0.5% NaOH (150 rpm for 2 h at 50 °C) or pre-treated with 1% H_2_SO_4_ (121 °C for 20 min in an autoclave plus 150 rpm for 2 h at 50 °C), and then the pre-treated pellets were washed with distilled water to neutral and used for sequential enzymatic hydrolysis with 150 rpm at 50 °C for 48 h. The loading dosage of mixed-cellulases (Imperial Jade Biotechnology Co., Ltd. Ningxia, China) was 10.60 FPU g^–1^ biomass and xylanase at 6.72 U g^–1^ biomass. The supernatants were collected to determine the yields of hexoses and pentoses released by enzymatic hydrolysis. All samples were carried out in triplicate.

### Bioinformatics

Protein transmembrane helices were predicted using TMHMM2.0 (http://www.cbs.dtu.dk/services/TMHMM-2.0/). Unrooted phylogenetic trees were constructed using MEGA6 with the neighbor-joining method and 1000 bootstrap replicates. Alignment of protein sequences was performed using ESPript 3.0 (http://espript.ibcp.fr/ESPript/cgi-bin/ESPript.cgi).

## Results

### The *Osfc24* mutant is defective in mechanical strength and has altered cell wall composition

**Fig. 1.**
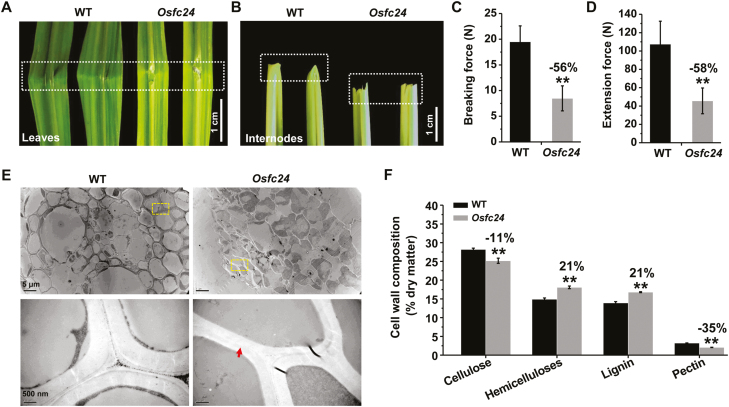
Phenotype of the rice *Osfc24* mutant. (A, B) Illustration of the brittleness of leaves and internodes in the *Osfc24* mutant relative to the wild-type (WT). The dashed boxes indicate the regions where breaks occurred. (C, D) Quantification of the breaking force and extension force of the internodes shown in (B). Data are means (±SD) of *n*≥20 plant main stems. (E) TEM images of sclerenchyma cell walls. The areas in the dashed boxes are magnified in the lower images. The arrow indicates an almost-missing middle lamella in the *Osfc24* mutant. (F) Cell wall composition of the stem tissues. Data are means (±SD) of three biological replicates. Significant differences between the WT and mutant were determined using Student’s *t*-test: **P<0.01. Percentage changes relative to the WT are indicated.

To identify the genes controlling plant mechanics and related cell-wall biosynthesis, we have previously generated a pool of rice brittleness mutants (Xie and Peng, 2011). The *fragile culm 24* (*Osfc24*) mutant was selected for this study from among the homozygous mutants. Compared to the wild-type (WT), we observed that the *Osfc24* mutant had fragile leaves and easily broken internodes (Fig. 1A, B). Accordingly, we found significantly reduced breaking and extension forces of stem tissues in the mutant compared with the WT (Fig. 1C, D; these being the major parameters that account for plant mechanical properties). We also observed reduced thickness in the sclerenchyma cells of *Osfc24* under TEM, and in particular the middle-lamella boundary was almost lost (Fig. 1E). Further chemical analysis indicated that the mutant had significantly reduced contents of cellulose and pectin (by 11% and 35%, respectively), but both hemicellulose and lignin levels were increased by 21% (Fig. 1F). These results therefore suggested that the brittleness phenotype of the *Osfc24* mutant may be mainly due to alterations in cell wall composition and wall thickness.

### The *Osfc24* mutant shows stunted growth with paler leaves

**Fig. 2.**
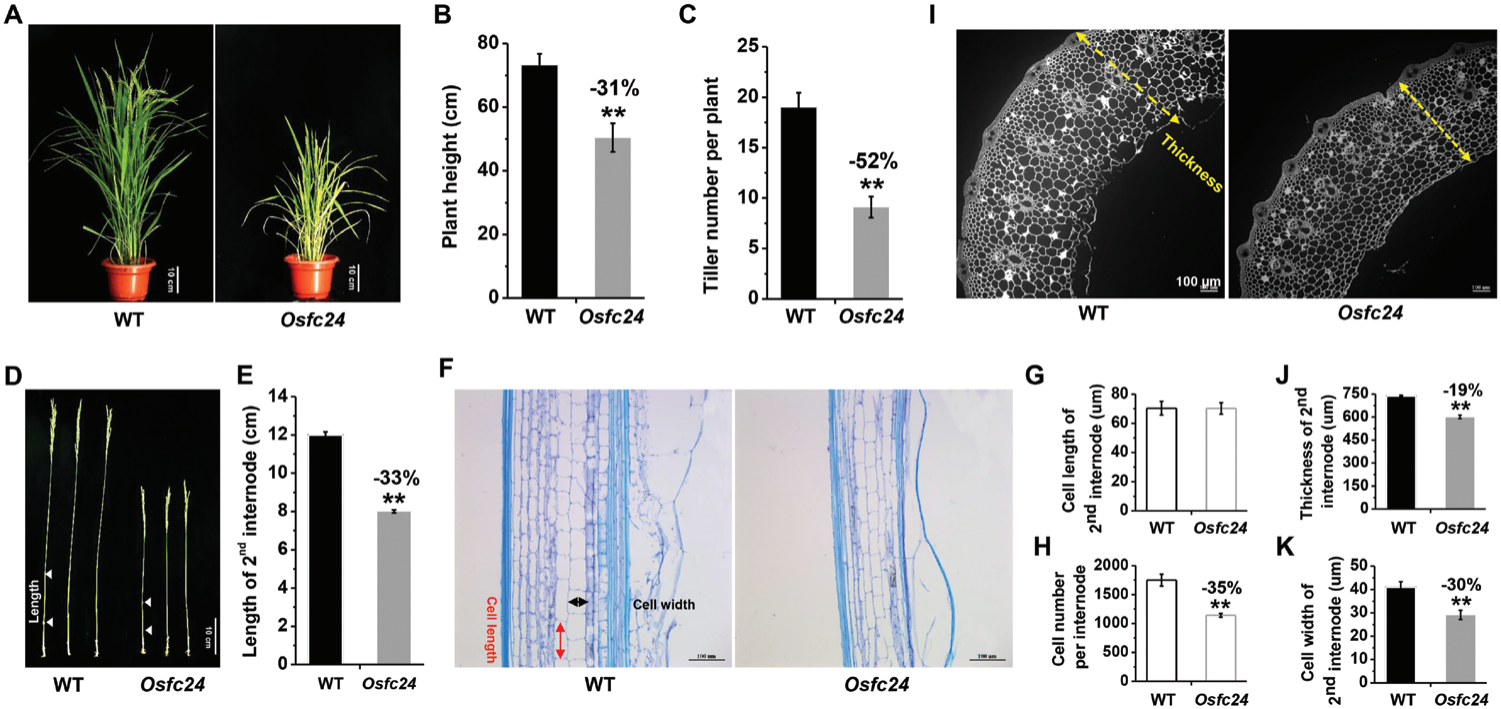
Characterization of cell growth in the rice *Osfc24* mutant. (A) The phenotypes of wild-type (WT) and *Osfc24* plants at the heading stage. (B) Plant height and (C) tiller numbers of plants at the heading stage. Data are means (±SD) of *n*≥20 replicates. (D) Images of whole stems and (E) the length of the 2nd internodes as illustrated in (D). Data are means (±SD) of *n*≥20 plant main stems. (F) Longitudinal sections of the 2nd internode. The measurements of cell length and width are indicated. (G) Cell length and (H) cell number of the 2nd internode as illustrated in (F). (I) Transverse section of the 2nd internodes as shown in (D), with the measurement of thickness indicated. (J) Quantification of stem thickness and (K) cell width of the 2nd internodes. Data are means (±SD) of *n*≥50 cells from sections of three plants (G, K) and *n*≥20 main stem internodes (H, J). Significant differences between the WT and mutant were determined using Student’s *t*-test: **P<0.01. Percentage changes relative to the WT are indicated.

The *Osfc24* mutant had leaves that were clearly paler than those of the WT throughout the full life cycle, and measurements at the heading stage indicated that the density of the green coloration was reduced by 32% (Fig. 2A, Supplementary Fig. S1). The plant height and tiller number of the mutant were also reduced by ~31% and ~52%, respectively (Fig. 2B, C), and the length of the 2nd internode was reduced by 33% compared (Fig. 2D, E). The cell length was similar between the mutant and the WT but the cell number per internode was decreased by 35% in the mutant (Fig. 2F–H). In the radial direction, we also observed significantly reduced thickness of the 2nd internodes and reduced cell width in the mutant (Fig. 2F, I–K). Taken together, the results indicated that the stunted growth of the *Osfc24* mutant was probably the consequence of both reduced cell number in the longitudinal direction and reduced cell width in the transverse direction.

### Map-based cloning of *Osfc24*

**Fig. 3.**
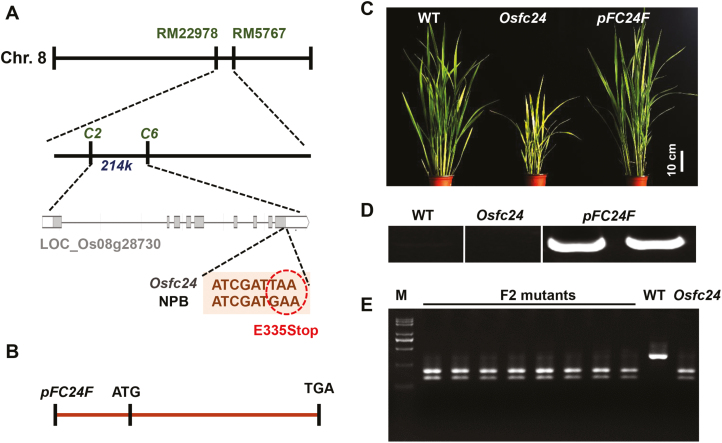
Map-based cloning and genetic complementation of the rice *Osfc24* mutant. (A) The mutant locus was mapped between molecular markers C2 and C6 in a 214-kb region. The candidate locus is *LOC_Os08g28730* (*FC24*). A nonsense mutation (E335Stop) is located in the 9th exon of *FC24*. (B) Schematic diagram of the *pFC24F* vector for the genetic complementation of the *Osfc24* mutant. (C) Phenotypes of 60-d-old wild-type (WT), *Osfc24*, and *pFC24F*-complemented plants. (D) Specific amplification of the *FC24* coding sequence in the genome DNA of WT, *Osfc24*, and *pFC24F* plants. (E) Co-segregation of F_2_ mutant plants using the restriction enzyme *Mse* I, a specific restriction site that was newly generated by the mutation of the *FC24* gene. The WT and the *Osfc24* mutant were used as negative and positive controls, respectively. M, marker.

Using a map-based cloning approach, we attempted to identify the gene of the *Osfc24* mutant. Based on a selection of 206 F_2_ mutant lines from a mapping population produced by crossing *Osfc24* and MH63 (a wild-type polymorphic *indica* variety), the mutant locus was mapped between markers RM22978 and RM5767 on chromosome 8. Fine-mapping with a larger population (~500 F_2_ mutant lines) allowed the region to be narrowed down to 214 kb between markers C2 and C6 (Fig. 3A). Sequencing of all candidate genes revealed a nonsense mutation (E335Stop) in the 9th exon of an ORF (LOC_Os08g28730) annotated as a NAD-dependent epimerase in the Rice Genome Annotation Project database (http://rice.plantbiology.msu.edu/).

To confirm this mutation as directly corresponding to the fragile-culm phenotypes, we carried out a genetic complementarity study by introducing the entire CDS region of LOC_Os08g28730 driven by its own promoter (a total of 3044 bp; named as *pFC24F*) into the background of the *Osfc24* mutant (Fig. 3B, Supplementary Table S1). The result was that the defects in mechanical strength, plant growth, and cell wall composition of the *Osfc24* mutant were almost completely rescued (Fig. 3C, Supplemental Fig. S2). To verify that the genetic complementary vector *pFC24F* had been transformed into the mutant, the *FC24* CDS was amplified by specific primers in the genome DNA of WT, *Osfc24*, and *pFC24F* plants, and this showed that the amplified sequence only existed in the *pFC24F* plants (Fig. 3D). As a result of the nonsense mutation of the *Osfc24* mutant, a new specific restriction enzyme site *Mse* I was produced. A co-segregation experiment of F_2_ mutant plants was therefore performed by single-enzyme digestion with *Mse* I, and this showed co-segregation between the brittleness phenotypes and the cleaved makers in the F_2_ population (Fig. 3E). The results therefore demonstrated that the mutation of LOC_Os08g28730 in the *Osfc24* mutant was responsible for the brittleness phenotype.

### *FC24*/*OsUGE2* results in high enzyme activity

**Fig. 4.**
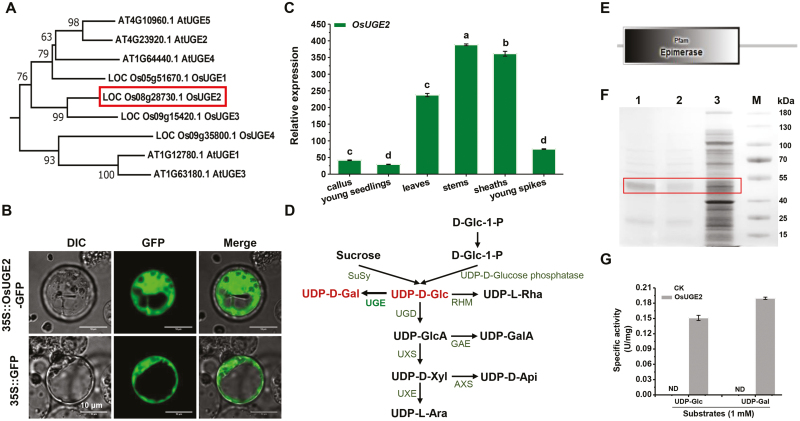
Expression, subcellular localization, and enzymatic activity assays of FC24/OsUGE2. (A) Unrooted tree of homologous UGE proteins in Arabidopsis and rice. (B) Subcellular location of OsUGE2. The images show rice protoplast cells expressing OsUGE2-GFP (upper) and GFP alone (lower). (C) Relative expression of *OsUGE2* in different tissues. *Ubiquitin* was used as the internal standard. Data are means (±SD) of three biological replicates. Different letters indicate significant differences between means as determined using ANOVA and LSD tests (*P*<0.01). (D) Biosynthetic pathway of UDP-sugar donors (modified from Caffall and Mohnen, 2009). (E) Prediction of OsUGE2 using SMART (http://smart.embl-heidelberg.de/) showed it to be a possible epimerase. (F) SDS-PAGE analysis of the recombinant OsUGE2 at each purification step. Samples were resolved on 12% polyacrylamide gel and then stained with Coomassie Blue R-250. Lane 1, purified OsUGE2 after desalting; lane 2, purified OsUGE2 after Ni-affinity column chromatography; lane 3, cellular proteins from the crude extract; M, markers. (G) The specific activity of purified OsUGE2 using substrates of UDP-Gal and UDP-Glc. CK was the control without the enzyme. ND, not detected. Data are means (±SD) of three biological replicates.

To examine the biological function of *FC24*, we performed a BLASTp search for homologous proteins in rice and Arabidopsis, and generated an unrooted phylogenetic tree by alignment of the proteins identified (Fig. 4A). The locus LOC_Os08g28730 belonged to the UGE family and we designed it as *OsUGE2*, which has been an uncharacterized UGE gene in rice and is separated into a different clade from other well-studied UGEs (e.g. OsUGE1; Guevara *et al.*, 2014). Multiple-sequence alignment revealed that the nonsense mutation (E335Stop) in the *Osfc24* mutant targets a conserved amino acid of OsUGE2 that is required for its function (Supplementary Fig. S3).

We then examined the subcellular localization OsUGE2. Because the TMHMM 2.0 server could not predict the transmembrane motif of the OsUGE2 protein (Supplementary Fig S4), we fused it with GFP under the control of the CaMV 35S promoter and transformed it into rice protoplasts. *OsUGE2::GFP* fluorescence was predominately observed in the cytoplasm (Fig. 4B), thus confirming its localization. We also observed that at the plant heading stage *OsUGE2* had much higher expression in the mature green tissues of the stems, sheaths, and leaves compared to the more immature tissues of the callus, young spikes, and young seedlings (Fig. 4C).

As UGE enzymes are characterized as producing UDP-Gal in plant pathways that interconvert nucleotide sugars (Fig. 4D), we examined the biochemical activity of OsUGE2 as an epimerase (Fig. 4E–G). Using a Pfam search (https://pfam.xfam.org/), OsUGE2 was predicted to encode an active epimerase (Fig. 4E). We then expressed OsUGE2 in *E. coli* as a His-fusion protein and assayed its enzyme activity *in vitro*. The recombinant protein was expressed and purified with a molecular mass of ~45 kDa, corresponding to its predicted molecular weight (Fig. 4F). UGE can interconvert UDP-Glc and UDP-Gal, and we measured remarkably high OsUGE2 enzyme activity *in vitro* using these two substrates, whereas the controls with no enzyme added did not show any epimerase activity (Fig. 4G). Our results therefore provided direct evidence that the OsUGE2 protein possessed epimerase activity for production of both UDP-Glc and UDP-Gal.

We then examined the expression levels of *UGE*s and their enzymatic activities in the *Osfc24* mutant. The expression levels *OsUGE1*, *OsUGE2*, and *OsUGE3* were significantly reduced in the *Osfc24* mutant compared with the WT, whilst those of *OsUGE4* and *OsPHD1* were increased (Supplementary Fig. S5). The net effect was that the overall *UGE* epimerase activity *in vivo* was significantly decreased in the *Osfc24* mutant (Supplementary Fig. S6). These results suggested that OsUGE1 and OsUGE3 may also participate in intracellular UDP-Gal supply, whereas OsUGE4 and OsPHD1 may function in specific tissue and cell secretion.

### The *Osfc24* mutant has reduced deposition of AGP

**Fig. 5.**
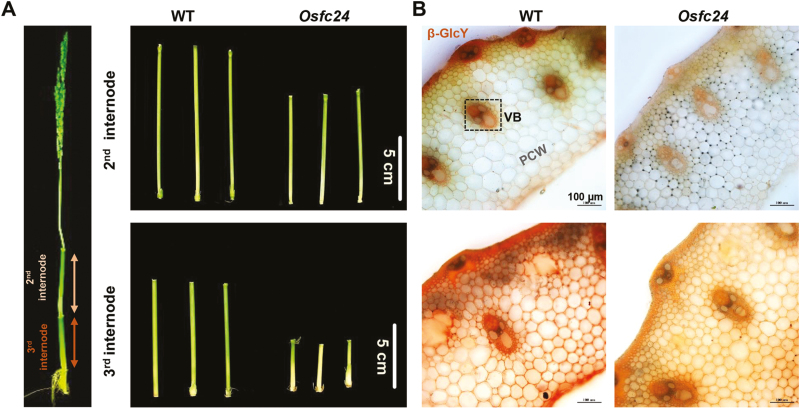
Yariv staining of arabinogalactan proteins (AGPs) in the internodes of rice wild-type (WT) and *Osfc24* mutant plants. (A) Illustration of the 2nd and 3rd internodes. (B) Staining of AGP with β-glucosyl Yariv reagent (β-GlcY) in cross-sections of the 2nd (upper) and 3rd (lower) internodes.

UDP-Gal is synthesized *de novo* by UGEs, and one of its pathways is for the biosynthesis of complex carbohydrates and glycoprotein into the cell wall. We therefore examine the monosaccharides of non-cellulosic polysaccharides of the cell walls in stem tissues. Compared with the WT, galactose and glucose were decreased by 27% and 24%, respectively, in the *Osfc24* mutant, but other sugars were not significantly altered (Table 1). AGPs are hydroxyproline-rich glycoproteins that enrich galactose and AG side-chains attached to the AGP core protein, and they react with Yariv reagent (β-GlcY; Yariv *et al.*, 1962). We utilized this to determine the localization of AGPs, and found that the *Osfc24* mutant clearly had reduced staining in both the 2nd and 3rd internodes of stem tissues (Fig. 5A, B), indicating reduced accumulation of AGPs in the cell walls compared with the WT.

**Table 1.** Composition of neutral monosaccharides in mature stems of the wild-type and the *Osfc24* mutant

	Rha	Fuc	Ara	Xyl	Man	Glc	Gal
Wild-type	0.04±0.01	0.01±0.01	8.80±1.73	83.74±1.28	0.04±0.01	5.63±0.65	1.75±0.02
*Osfc24*	0.03±0.00	0.01±0.00	8.90±1.05	85.45±0.75	0.03±0.00	4.29±0.44^**^	1.28±0.06^**^

Data are means (±SD) of three biological replicates. Significant differences between the wild-type and the mutant were determined using Student’s *t*-test: ** *P*<0.01.

**Fig. 6.**
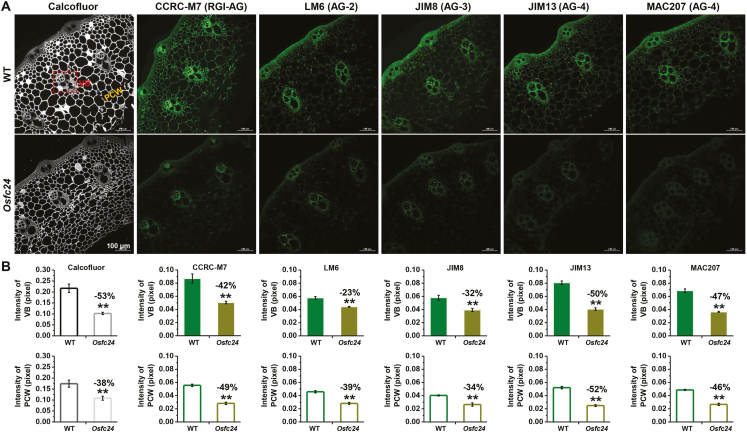
*In situ* immunolabeling of arabinogalactan (AG) in the internodes of rice wild-type (WT) and *Osfc24* mutant plants. (A) Immunofluorescent labeling of the 2nd internode using monoclonal antibodies directed at cell wall glycan. Calcofluor (white) stains the cell wall (β-glucans). LM6, JIM8, JIM13, MAC207, and CCRC-M7 are antibodies against AG. VB, vascular bundle; PCW, parenchyma cell wall. (B) Fluorescence intensities of the VB and PCW regions as illustrated in (A). Data are means (±SD) of *n*≥10 sections in each of three biological replicates. Significant differences between the WT and mutant were determined using Student’s *t*-test: **P<0.01. Percentage changes relative to the WT are indicated.

We also conducted immunolabeling assays with several anti-AG mAbs, namely LM6 (for 1, 5-Ara linkage AGPs and pectin), JIM8 (for AG), JIM13 (for AG and AGPs), MAC207 (AGPs), and CCRC-M7 (for a galactan epitope found in AGPs and RG I). In general, the *Osfc24* mutant showed relatively fainter fluorescence than that of the WT for all five of the anti-AG mAbs (Fig. 6A). In particular, semi-quantitative analysis of the images indicated a significantly reduced fluorescence intensity in the vascular bundles and parenchyma cell walls of the mutant (Fig. 6B). The results were consistent with the reduced accumulation of AGP observed in the cell walls of the *Osfc24* mutant.

### The *Osfc24* mutant has reduced levels of galactolipids and reduced photosynthetic activity

**Fig. 7.**
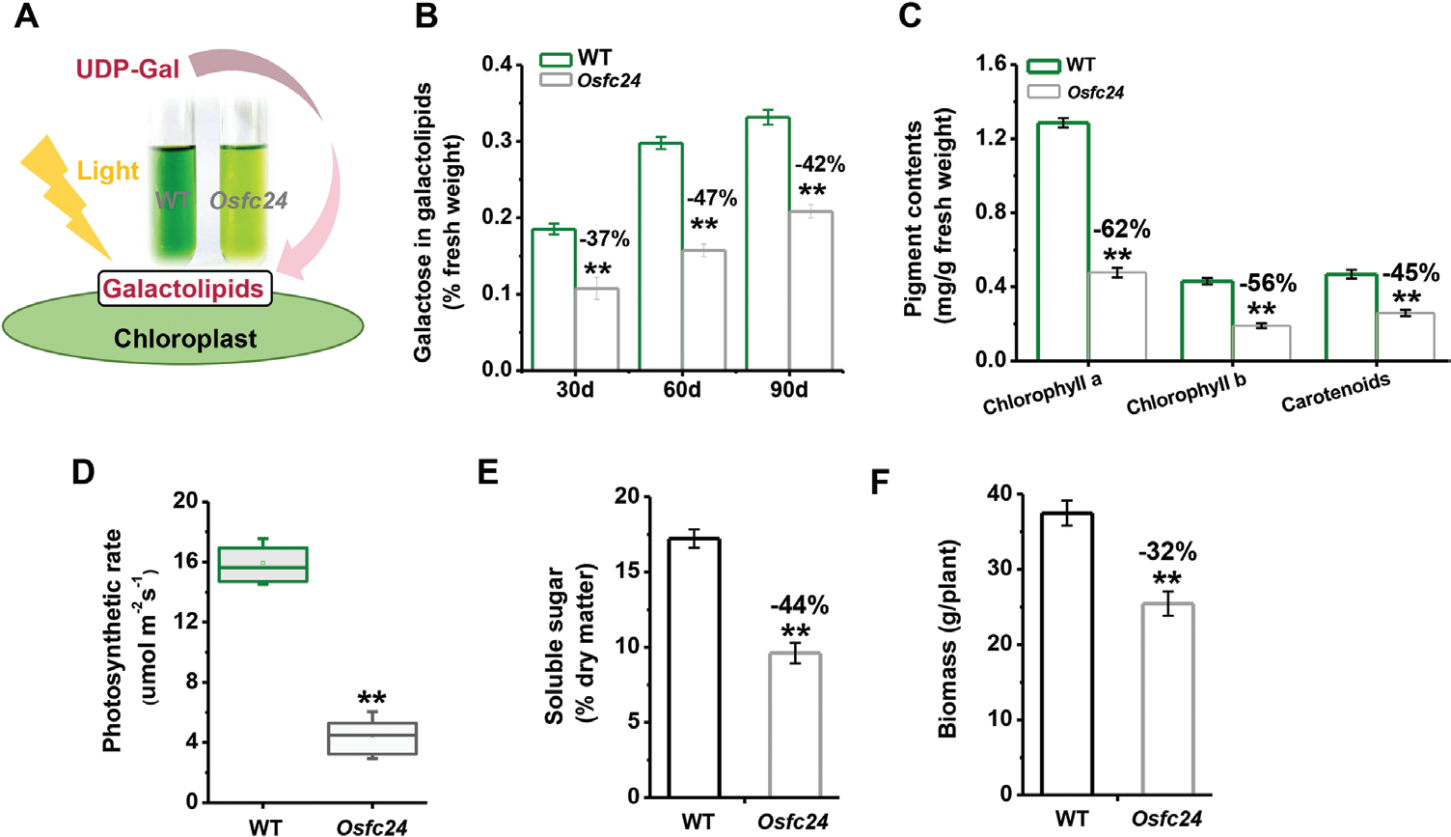
Characterization of photosynthesis in rice wild-type (WT) and *Osfc24* mutant plants. (A) Leaf extracts show defects in the chloroplasts of the mutant, which may derive from a lack of flux of UDP-Gal into the galactolipids of the chloroplasts when the plants are engaged in photosynthesis. (B) Quantification of galactoses released from galactolipids for plants of different ages. (C) Contents of chloroplast pigments and (D) photosynthetic rate of leaves of 60-d-old plants, (E) content of soluble sugars of mature stems, and (F) biomass of mature plants. Data are means (±SD) of either three replicates (B, C, E), *n*≥12 replicates (D; the uppermost leaves were measured), or *n*≥20 mature plants (F). Significant differences between the WT and mutant were determined using Student’s *t*-test: **P<0.01. Percentage changes relative to the WT are indicated.

Because UDP-Gal has been characterized as being involved in galactolipid biosynthesis in the chloroplasts (Li *et al.*, 2011) and based on our observation of extracts of mature leaves of the *Osfc24* mutant being paler those of the WT (Fig. 7A), we deduced that the mutant may also have been defective in galactolipids. As plants aged, we found a consistent reduction in galactose levels derived from galactolipid in the mutant (Fig. 7B), suggesting that OsUGE2 participates in galactolipid biosynthesis. As galactolipids are the main components of chloroplasts, accounting for 20% of their dry matter, we measured the pigment contents of mature leaves. Compared to the WT, the *Osfc24* mutant showed dramatically decreased concentrations of chlorophyll *a*, chlorophyll *b*, and carotenoids, by 62%, 56%, and 45%, respectively (Fig. 7C). To examine the potential impact of this disruption of the chloroplasts, we used combined gas-exchange and fluorescence approaches to measure three important parameters of photosynthesis, and found that the photosynthetic rate, stomatal conductance, and transpiration rate were all consistently reduced in the mutant (Fig. 7D, Supplementary Fig. S7). In addition, we found that the stems of the mutant plants had significant reductions in soluble sugar content and biomass of 44% and 32%, respectively, compared with the WT (Fig. 7E, F). Taken together, the results suggested that *OsUGE2* was positively associated with galactolipid biosynthesis and plant photosynthesis, with its mutation thus leading to stunted growth and paler leaves.

### The *Osfc24* mutant has defects cellulose properties and disrupted orientation of microfibrils

**Fig. 8.**
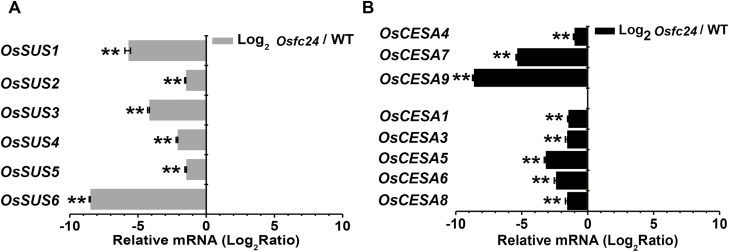
Gene expression of the rice *Osfc24* mutant relative to the wild-type (WT), as determined by qRT-PCR. (A) Fold-change in expression of sucrose synthase genes (*SUS*s). (B) Fold-change in expression of cellulose synthase genes (*CESA*s). Data are means (±SD) of three biological replicates. Significant differences compared to (*Osfc24*/WT)=1 (i.e. log_2_ ratio of zero) were determined using Student’s *t*-test: **P<0.01.

**Fig. 9.**
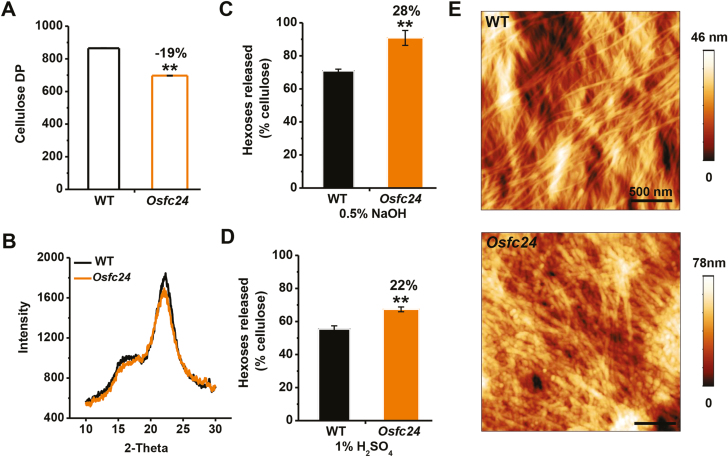
Characterization of cellulose properties in rice wild-type (WT) and *Osfc24* mutant plants. (A) Crude cellulose degree of polymerization (DP) of mature stem tissues. (B) Cellulose crystalline index (CrI) of mature stem tissues as determined by intensity of X-ray diffraction. (C, D) Yield of hexoses released from enzymatic hydrolysis following pre-treatment with (C) 0.5% NaOH and (D) 1% H_2_SO_4_. Values are expressed as % of total dry mass. (E) Cellulose microfibrils as observed using atomic force microscopy. Data are means (±SD) of three biological replicates. Significant differences between means were determined using Student’s *t*-test: **P<0.01. Percentage changes relative to the WT are indicated.

Since photosynthesis is tightly associated with carbon fluxes used for cellulose production (Boex-Fontvieille *et al.*, 2014), we assumed that the defects in cellulose that we observed in the *Osfc24* mutant may have been partially due to the reduced capacity of carbon supply. Sucrose synthase (SUS) enzymes plays a key role in carbohydrate metabolism and cellulose biosynthesis (Jiang *et al.*, 2012; Fan *et al.*, 2017). All six *OsSuS* genes that we examined were significantly down-regulated in the *Osfc24* mutant compared with the WT (Fig. 8A). In addition, we found that eight cellulose synthase (*OsCESA*) genes also had much lower expression in the mutant (Fig. 8B), and this was consistent with the reduced cellulose levels (Fig. 1F). The degree of polymerization (DP) of β-1, 4-glucans was significantly reduced in the mutant (Fig. 9A), probably due to the defects in cellulose biosynthesis (Li *et al.*, 2017). We also detected a relatively reduced cellulose crystalline index (CrI) in the mutant (Fig. 9B), which may have been largely attributable to reduced cellulose levels and increased hemicelluloses (Xu *et al.*, 2012). As cellulose DP and CrI are crucial factors that account for the recalcitrance of lignocellulose, we examined biomass enzymatic digestibility by calculating the yield of hexoses released from cellulase enzyme hydrolysis following mild alkali or acidic pre-treatments. The *Osfc24* mutant showed much higher yields of hexoses than the WT (Fig. 9C, D), which was consistent with previous findings that reduced cellulose DP and CrI can enhance biomass enzymatic digestibility following various physical and chemical pre-treatments (Wu *et al.*, 2013; Zhang *et al.*, 2013).

We also examined the orientation of cellulose microfibrils using atomic force microscopy. Following treatment with 8% acidic chlorite, the cellulose microfibrils could be clearly viewed at the nano-scale in parenchyma-type secondary walls (Fig. 9E). The WT showed a poly-lamellate structure, with the cellulose microfibrils oriented in a common direction in each layer and the orientation between adjacent lamellae differed by 30–90°, which was similar to a previous report (Zhang *et al.*, 2017). In contrast, in the mutant the microfibrils exhibited a broken and disordered pattern with a rough surface, which was consistent with the reduced cellulose DP. Overall, the mutation of OsUGE2 caused defects in the levels and properties of cellulose, and also altered the orientation of the microfibrils, which combined to affect the properties of the cell walls.

## Discussion

### OsFC24/UGE2 is a newly identified protein with novel biological functions

**Fig. 10.**
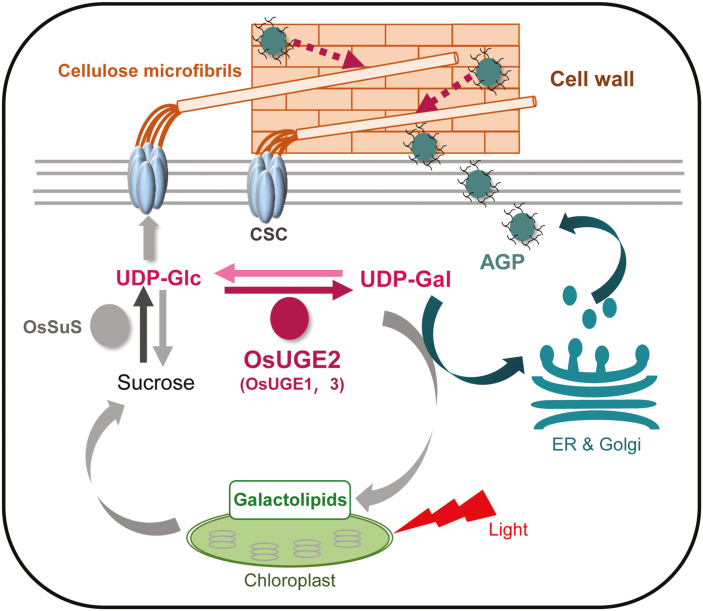
A proposed model for how OsUGE2 participates in different metabolic pathways to co-modulate cellulose biosynthesis and wall assembly by dynamically providing UDP-Gal and UDP-Glc substrates. OsUGE2 (probably in conjunction with OsUGE1 and OsUGE3) synthesizes UDP-Gal *de novo* by interconverting UDP-Glc, and this supplies the biosynthesis of arabinogalactan protein (AGP) and galactolipids in the chloroplasts. Accumulation of AGP may positively affect cellulose synthesis and hence plant mechanics. Galactolipids are an essential component of the chloroplasts for photosynthesis, and hence help to increase the accumulation the carbon source (sucrose) that provides the UDP-Glc substrate for cellulose biosynthesis. Solid lines indicate metabolic flows and dashed lines indicate potential functions. CSC, cellulose synthase complex; ER, endoplasmic reticulum; OsSuS, rice sucrose synthase.

Characterization of plant mutants with mechanical defects provides an effective approach to explore cell wall biosynthesis and related biological functions. Several distinct mutants have already been well characterized (Zhang and Zhou, 2011), and our current study identified a novel rice fragile-culm *Osfc24* mutant, encoding a cyto-localized protein, OsUGE2. The UGE family usually contains only a few members, such as five isoforms in Arabidopsis (AtUGE1–5) and four in rice (OsUGE1–4), but despite this most of the UGE proteins in rice have not been well studied (Seifert *et al.*, 2002; Barber *et al.*, 2006; Rösti *et al.*, 2007; Kim *et al.*, 2009; Guevara *et al.*, 2014). Although all UGEs are catalytically active in both biosynthetic reactions for UDP-Glc and UDP-Gal at the biochemical level, each individual isoform may have its own characteristic metabolism process *in vivo*. In this study, we found that OsUGE2 participated in the biosynthesis of both AGPs and galactolipids in the cytoplasm of stem and leaf tissues (Figs 5–7), which is in contrast to OsPHD1, which is reported as only being involved in galactolipid biosynthesis in leaf chloroplast (Li *et al.*, 2011). In addition, we found that OsUGE2 had co-suppressed transcriptional levels of *OsUGE1* and *OsUGE3* in the *Osfc24* mutant (Supplementary Fig. S5), which may have contributed to the severe phenotype (Figs 1, 2). It therefore seems that OsUGE2 may cooperate with OsUGE1and OsUGE3 to supply UDP-Gal substrates in the cytoplasm. Consequently, we deduce that the OsUGE2 may participate in two distinct metabolism pathways by dynamically providing the UDP-Gal substrate, as illustrated in the proposed model shown in Fig. 10.

### AGP accumulation may affect mechanical properties in the *Osfc24* mutant

AGPs consists of arabinogalactan glycans that are *O*-glycosylated on a core-protein backbone and they are mainly deposited into cell walls. It has been assumed that AGPs may play a role in maintaining plant strength by their interactions with pectin as an adhesion molecule (Tan *et al.*, 2013). Based on immunolabelling and histological staining (Figs 5, 6), we found that AGP accumulation in the cell walls of the *Osfc24* mutant was much reduced, suggesting that the mutation of OsUGE2 may have resulted in less UDP-Gal substrate being provided for the biosynthesis of AGPs. Staining with Yariv reagent indicated that AGPs were mainly enriched in the sclerenchyma cells and the *Osfc24* mutant had significantly reduced breaking and extension forces, and hence our study confirmed previous findings that AGPs may contribute to plant mechanical strength by directly or indirectly regulating cellulose deposition (Casero *et al.*, 1998; Loopstra *et al.*, 2000; Ito *et al.*, 2005; Zhou *et al.*, 2009). To further examine the impact of AGPs on cellulose biosynthesis and deposition, we analysed *OsCESA*s following treatment with Yariv reagent (β-GlcY, 50 uM for 5 d), and found that expression levels of both primary and secondary wall *OsCESA*s were severely reduced (Supplementary Fig. S8). Hence, we deduce that the disrupted orientation of the cellulose microfibrils in the *Osfc24* mutant may be partially due to the lower accumulation of AGP (Fig. 10). However, further studies are needed to provide direct evidence that AGP accumulation can control the orientation of the microfibrils.

### OsUGE2 affects plant photosynthesis via galactolipid production

Plant galactolipids are the most abundant lipids of chloroplast membranes (Shimojima and Ohta, 2011), and the well-developed thylakoid membranes determine photosynthetic reactions in higher plants. Consequently, galactolipids are thought to play an important role in the organization of photosynthetic membranes (Benning, 2008). For example, Arabidopsis and rice mutants defective in galactolipids have fewer chlorophyll contents, inhibited photosynthetic activity, and abnormal plant growth (Kelly *et al.*, 2003; Kobayashi *et al.*, 2007). In this study, we also detected significantly reduced pigment content (Figs 2A, 7, Supplementary Fig. S1), photosynthetic rate (Fig. 7D), and photosynthetic products (Fig. 7E, F) in the *Osfc24* mutant, providing solid evidence that OsUGE2 plays an important role in photosynthesis by providing UDP-Gal for the biosynthesis of galactolipids.

### OsUGE2 affects cellulose biosynthesis and recalcitrance of lignocellulose

Cellulose is the important loading-bearing skeletal component in plant cell walls. It comprises long, rigid microfibrils and contains both ordered crystalline regions and disordered or non-crystalline regions (McFarlane *et al.*, 2014). SUS is the key enzyme for regulating carbon flux by providing the UDP-Glc substrate for cellulose biosynthesis (Boex-Fontvieille *et al.*, 2014; Fan *et al.*, 2017; Ivakov *et al.*, 2017). We found that numerous *SUS*s and *CESA*s were significantly down-regulated in the *Osfc24* mutant (Fig. 8), and hence we conclude that the inhibition of photosynthesis may have affected sucrose synthesis, thus resulting in less provision of the UDP-Glc substrate for cellulose biosynthesis in the mutant (Fig. 10). In addition, we also observed alterations in cellulose properties and structure in the *Osfc24* mutant (Fig. 9) that confirmed that the mutation of OsUGE2 could affect cellulose biosynthesis. We further found that the expression levels of both primary and secondary wall *OsCESA*s were severely reduced by treatment with Yariv reagent (Supplementary Fig. S8), which suggested that both photosynthesis and AGP accumulation could affect cellulose biosynthesis. Taken together, we speculate that OsUGE2 may regulate cellulose biosynthesis and structure via two distinct metabolism pathways. However, whether the mutation of OsUGE2 directly leads to the reduction in UDP-Glc substrate or whether it acts through an indirect pathway to reduce cellulose production requires further study.

It has been shown that a defect in cellulose biosynthesis in the rice *CesA* mutant can negatively affect cellulose properties (DP and CrI) and alter the microfibril orientation, thereby affecting the cell wall properties (Li *et al.*, 2017). We also found reductions in cellulose DP and CrI in the *Osfc24* mutant together with significantly enhanced biomass enzymatic saccharification following chemical pre-treatments (Fig. 9A), which were consistent with previous studies (Wu *et al.*, 2013; Li *et al.*, 2017). Hence, our work has clearly demonstrated that the mutation in OsUGE2 considerably affects cellulose biosynthesis, leading to reduced lignocellulose recalcitrance.

### Conclusions

We have demonstrated that OsUGE2 participates in distinct metabolic pathways to co-modulate cellulose biosynthesis and wall assembly by dynamically providing UDP-Gal and UDP-Glc substrates (Fig. 10). The mutation of OsUGE2 in *Osfc24* plants also decreased accumulation of AGPs into the cell wall and reduced the galactolipid content in chloroplasts, the integrated effect of which affected cell wall properties and biomass production in the mutant. This study therefore provides insights into plant cell wall formation and photosynthesis in rice that will have relevance in other species.

## Supplementary data

Supplementary data are available at *JXB* online.

Table S1. Primers for map-based cloning of *Osfc24*.

Table S2. Primers used for functional studies of *OsUGE2*.

Table S3. Primers used for qRT-PCR of *OsSUS*s and *OsCESA*s.

Fig. S1. Quantification of green leaf color in the *Osfc24* mutant and wild-type.

Fig. S2. Genetic complementation test of brittleness properties in the *Osfc24* mutant using *pFC24F.*

Fig. S3. Sequence alignments of the UGE protein family in rice and Arabidopsis.

Fig. S4. Prediction of the OsUGE2 protein domain using the TMHMM2.0 program.

Fig. S5. Transcription levels of *OsUGE*s and *OsPHD1* in stem tissues of the *Osfc24* mutant and the wild-type.

Fig. S6. UGE activity in stem tissues of the *Osfc24* mutant and the wild-type.

Fig. S7. Stomatal conductance and transpiration rate in the *Osfc24* mutant and the wild-type.

Fig. S8. Transcription levels of *OsCESA*s in young root tissues of the wild-type following treatment with Yariv reagent.

eraa044_suppl_supplementary_tables_S1_S3_figures_S1_S8
