# Supplementary material for: A novel rice fragile culm 24 mutant encodes a UDP-glucose epimerase that affects cell wall properties and photosynthesis
Source: J Exp Bot. 2020 Feb 17;71(10):2956–69. doi: 10.1093/jxb/eraa044 (PMC7260720; doi:10.1093/jxb/eraa044)

A novel rice *fragile culm 24* mutant encodes a UDP-glucose epimerase that affects cell wall property and photosynthesis

Ran Zhang[1], Huizhen Hu[2], Youmei Wang[1], Zhen Hu[1], Shuangfeng Ren[1], Jiaying Li[1], Boyang He[1], Yanting Wang[1], Tao Xia[1,3], Peng Chen[1], Guosheng Xie[1], Liangcai Peng[1*]

[1] Biomass & Bioenergy Research Centre, College of Plant Science & Technology, Huazhong Agricultural University, Wuhan 430070, China.

[2] State Key Laboratory of Biocatalysis & Enzyme Engineering, College of Life Science, Hubei University, Wuhan 430062, China.

[3] College of Life Science & Technology, Huazhong Agricultural University, Wuhan 430070, China.

Supplemental Table S1. Primers for map-based cloning of *Osfc24*.

Primer name	Primer sequence (5’ to 3’)
RM22978-F	ACACGTTTGTACGTTCGTTTCG
RM22979-R	GCCGACAAACACTCTCATTGG
RM5767-F	GTCCGACTCACCTCCCTCTTGC
RM5768-R	CCTCATCCTCTCCACGCTCTCC
C2-F	CAGCAATAAGATGCTACAAGAA
C2-R	GAAAACGAAATAAACAAACTCC
C6-F	AGCCACCGACCAAATAATCAG
C6-R	GGACGGCGGAAGTATCTCAAT

Supplemental Table S2. Primers for functional studies of *OsUGE2*.

Primer name	Primer sequence (5' to 3')	Purpose
Promoter-UGE2-F	ATTGAAAGATGAAGACAGGGGAC	Promoter cloning of *OsUGE2*
Promoter-UGE2-R	GAATGTGGGGAGGAGAAGAGTAG	
CDS-UGE2-F	CCTGAATCCCTCCTCCTACTCT	CDS region cloning of *OsUGE2*
CDS-UGE2-R	CCCGGGAAGAGAAAACATGAGCATCGGT	
Q-OsUGE1-F	TACTGCTCCGATACTTCAACCC	Q-PCR of *OsUGEs*
Q-OsUGE1-R	CCATCCGCTAGATCAACAACAT	
Q-OsUGE2-F	TATGGATACGGGTCACTTGACTC	
Q-OsUGE2-R	ACCAGTCCATATCCGTTGTGAC	
Q-OsUGE3-F	TGGAACAGGAAAGGGAACATC	
Q-OsUGE3-R	TCGTGGACCAATAACCAAAGG	
Q-OsUGE4-F	TCGCTACTCCTGACATTGGTT	
Q-OsUGE4-R	TGATCGCCCTAATTCTGCTC	
Q-OsPHD1-F	CTGTGACAATAAGCCTCTTTCCAG	
Q-OsPHD1-R	GCTCCCAACCAATCTCAGAAC	
Mse I-UGE2-F	TGGTGATGCGGAGATT	Amplify the fragment covered the mutation site of *Osfc24*
Mse I-UGE2-R	CGTTATGCTTGGCAGAGT	
GFP-UGE2-F	CAGTGGTCTCACAACATGGCGGTGGAGAAGACGGT	Subcellular localization of OsUGE2
GFP-UGE2-R	CAGTGGTCTCATACAGTGGAAGTGGCCGTTGCCGT	
28a-OsUGE2-F	CGGGATCCATGGCGGTGGAGAAGACG	Recombinant expression of OsUGE2 in *E. coli.*
28a-OsUGE2-R	CCCAAGCTTGTGGAAGTGGCCGTTGC	

Supplemental Table S3. Primers for qRT- PCR of *OsSUSs* and *OsCESAs*.

Primer name	Primer sequence (5' to 3')
Q-SUS1-F	CATCTCAGGCTGAGACTCTGA
Q-SUS1-R	CAAATTCAATCGACCTTACTT
Q-SUS2-F	GAGGCTGATGACCTTGACTGG
Q-SUS2-R	CCCTCCATTACTTGGATGTGCT
Q-SUS3-F	GTGAGACACGCCGCTACCTGGAGA
Q-SUS3-R	CCCTGAAATCAACTAAACCCT
Q-SUS4-F	TGAACTGGCGAAGACTGTACC
Q-SUS4-R	CAATGGCAATGAAGCACTGAG
Q-SUS5-F	CAGGTTAGCCGGGCAGAAG
Q-SUS5-R	CATTGTACACAAATCTCACGCAAG
Q-SUS6-F	CGAACAACCAGCACAACCTAC
Q-SUS6-R	GGAGCAGTGGTCCAAGTAAGT
Q-CESA1-F	GGCATGGTGGCGGGTATAT
Q-CESA1-R	AGGCGACGGCTTTCTGAGT
Q-CESA3-F	GTGTAGATAGAAACACGAAAGGTG
Q-CESA3-R	CAATGGGCACCCACACACGT
Q-CESA4-F	CCATCGTCTACCCGTTCACC
Q-CESA4-R	TCCAGCCCAGCAATCATCTT
Q-CESA5-F	CCTTGTTAATACCCCCTACC
Q-CESA5-R	AATCCAAACCACACTCCTCC
Q-CESA6-F	GAAGGCAAAACAGCACACCCA
Q-CESA6-R	CCAAGTAAGCTCAGCGATGA
Q-CESA7-F	TTAACGGGGTTCAAGATGC
Q-CESA7-R	ATGGTGTTGGTGTAGGCGA
Q-CESA8-F	TGGTCGGTGTTGTTGCTGG
Q-CESA8-R	ATGGTCGGAGTGCGGTTTT
Q-CESA9-F	TACAAGAACGGCAACCTCA
Q-CESA9-R	AAGAACAAACTCGCAAACG
Q-UBQ-F	CCAGGACAAGATGATCTGCC
Q-UBQ-R	AAGAAGCTGAAGCATCCAGC

Supplemental Figure Legends

Supplemental Figure S1. Leaf green-color quantification of *Osfc24* mutant. (A) Leaf phenotype at heading stage. (B) Color quantification. Error bars represent mean ± SD (three biological replicates) with Student's *t*-test for significant difference, n ≥ 10 leaves in were measured in each replicate. The rate (%) calculated by subtraction of values between the WT and *Osfc24* divided by the WT value. Scale bar indicates 2 cm in (A).

Supplemental Figure S2. Genetic complementation test of rice brittleness mutant *Osfc24* by *pFC24F*. (A) Plant height; (B) Tiller number; (C) Breaking force; (D) extension force of 2nd internode at heading stage (E) Cell wall compositions of mature plants of wild type (WT) and *pFC24F*. Error bars represent mean ± SD (three biological replicates) with Student's t-test for significant difference, n ≥ 20 plants in (A-D) were measured in each replicate.

Supplemental Figure S3. Protein sequence alignment of the UGE protein family in rice and *Arabidopsis*. Green asterisk represents the mutated amino acid site.

Supplemental Figure S4. Prediction of OsUGE2 protein domain by TMHMM2.0 program.

Supplemental Figure S5. Transcriptional analysis of *OsUGEs* and *OsPHD1* genes in stems tissues of the *Osfc24* mutant. *Ubiquitin* gene used as internal standard. Error bars represent mean ± SD (three biological replicates). ***P* < 0.01 by Student's t-test.

Supplemental Figure S6. UGE activity in stem tissues of the *Osfc24* mutant. Error bars represent mean ± SD (three biological replicates). ***P* < 0.01 by Student's t-test. The rate (%) calculated by subtraction of values between the WT and *Osfc24* divided by the WT value.

Supplemental Figure S7. Stomatal conductance and transpiration rate in the *Osfc24*

mutant. Values represent means ± SD, n = 12. ***P* < 0.01 by t-test.

Supplemental Figure S8. Transcriptional analysis of *OsCESAs* in young root tissues of the WT under Yariv treatment. (A) Germinated seeds were treated with β-glucosyl Yariv reagent (50 μM) for 5 days. (B) Fold of change on *OsCESAs* after Yariv treatment. *Ubiquitin* gene used as internal standard. Error bars represent mean ± SD (three biological replicates). ***P* < 0.01 by Student's t-test.

Zhang *et al*., Supplemental Figure S1

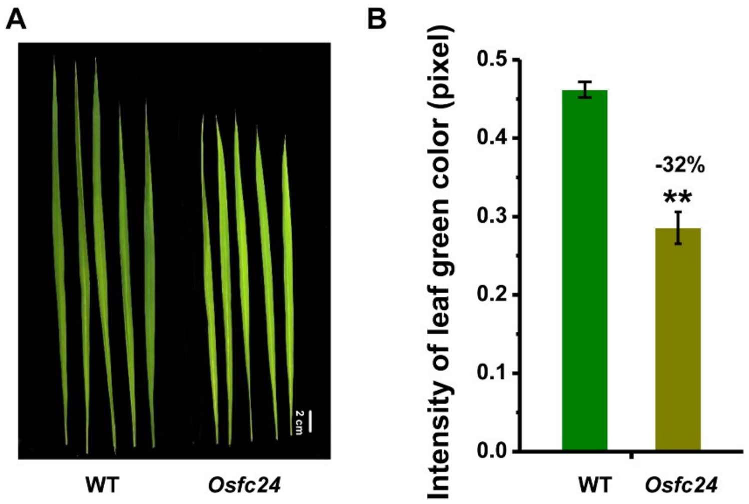

Zhang *et al*., Supplemental Figure S2

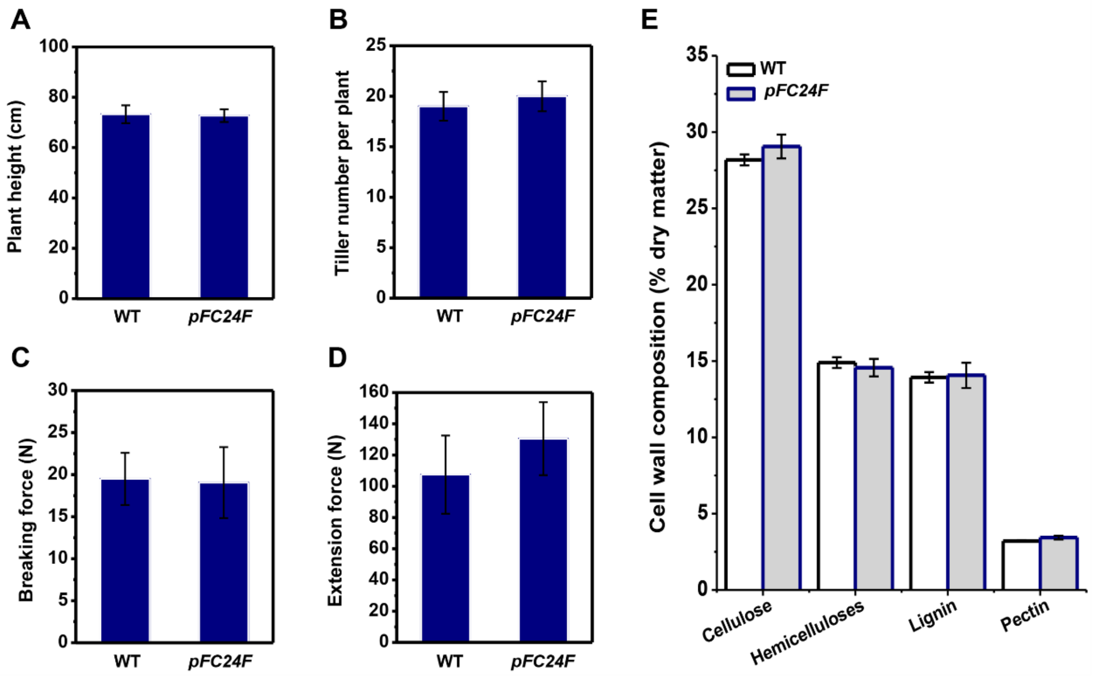

Zhang *et al.*, Supplemental Figure S3

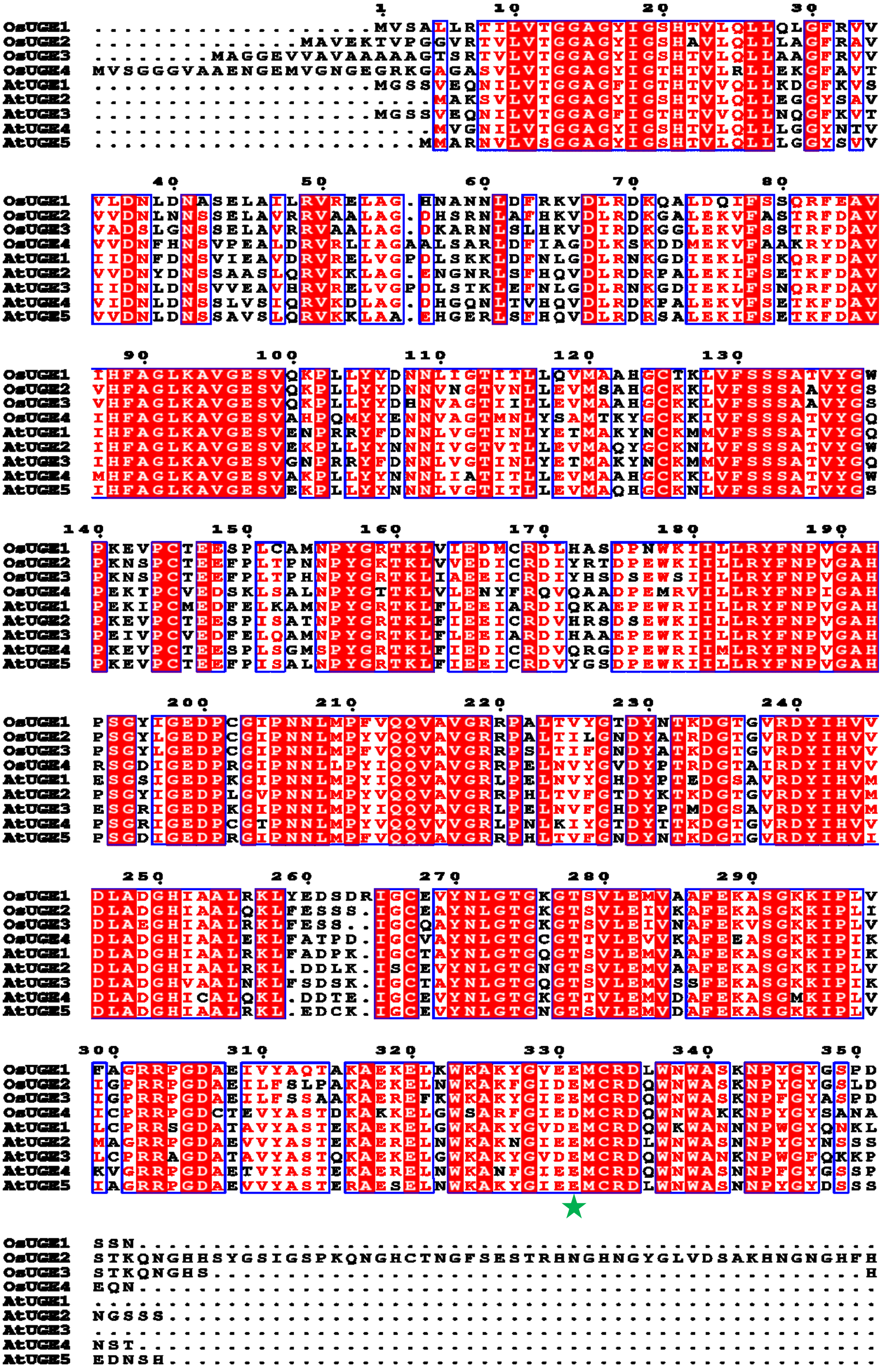

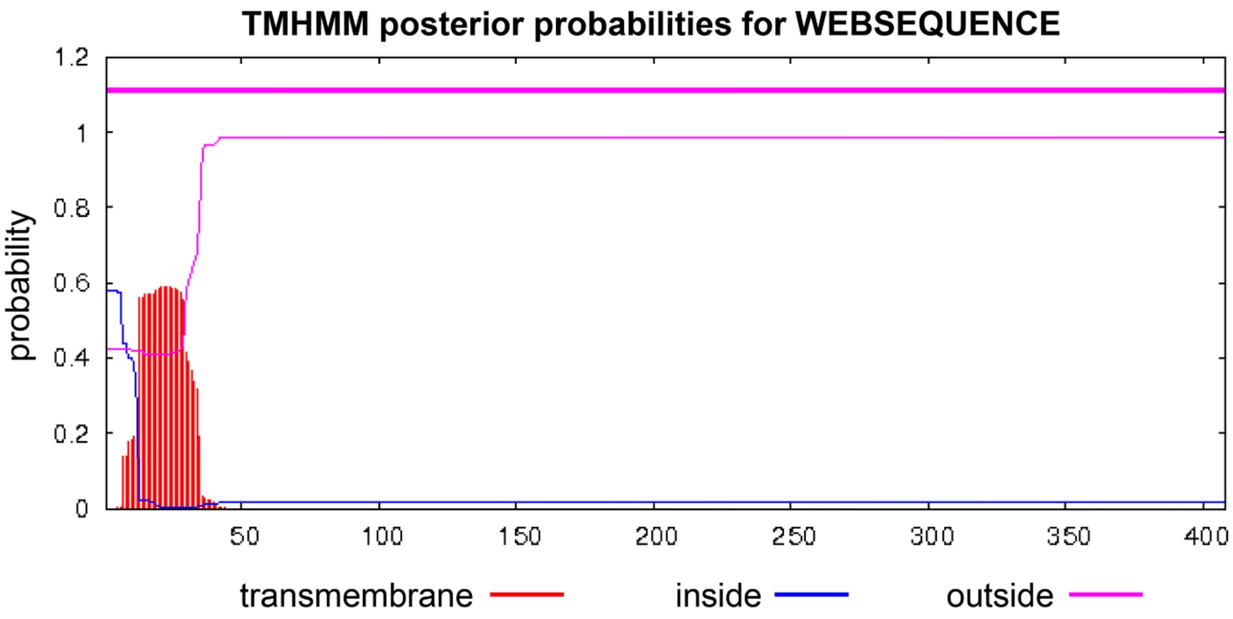
TMHMM posterior probabilities for WEBSEQUENCE
1.2
1
0.8
0.6
0.4
0.2
0
probability
50
100
150
200
250
300
350
400
transmembrane
inside
outside

Zhang *et al.*, Supplemental Figure S5

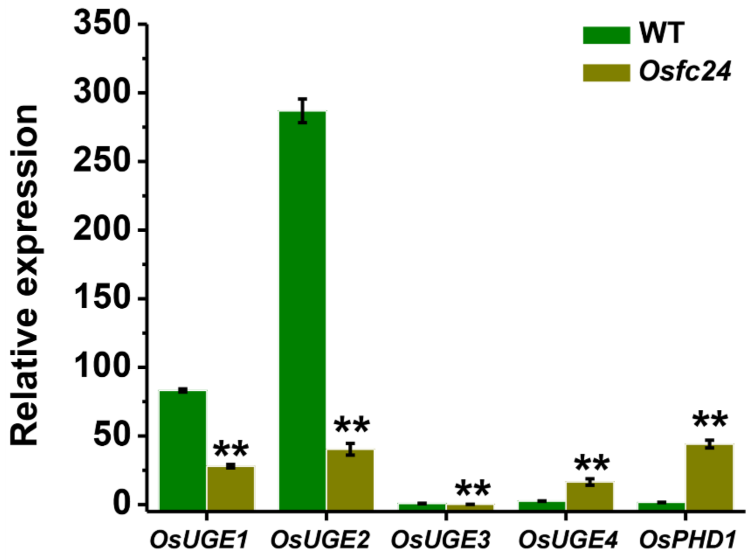

Zhang *et al.*, Supplemental Figure S6

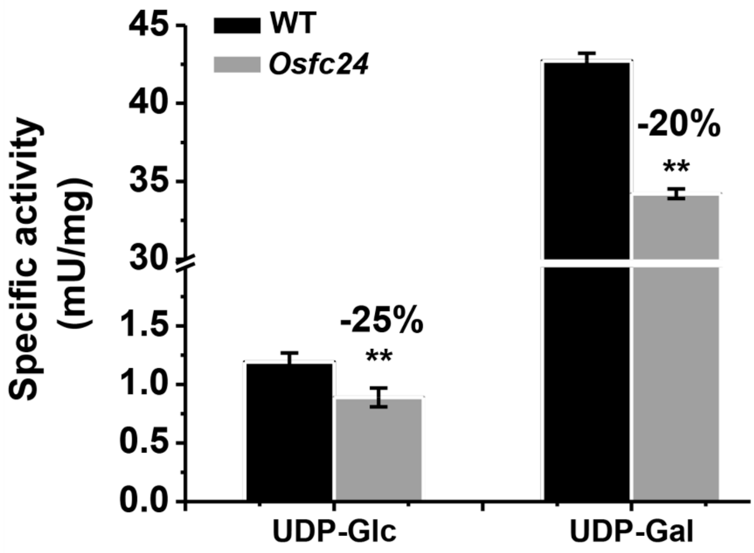

Zhang *et al*., Supplemental Figure S7

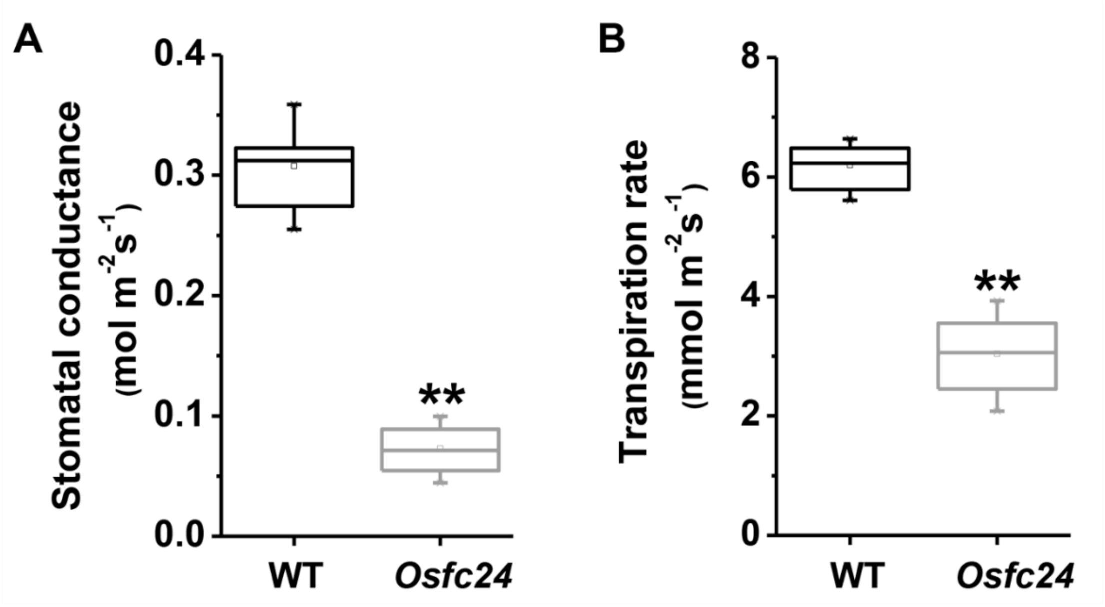

Zhang *et al.*, Supplemental Figure S8

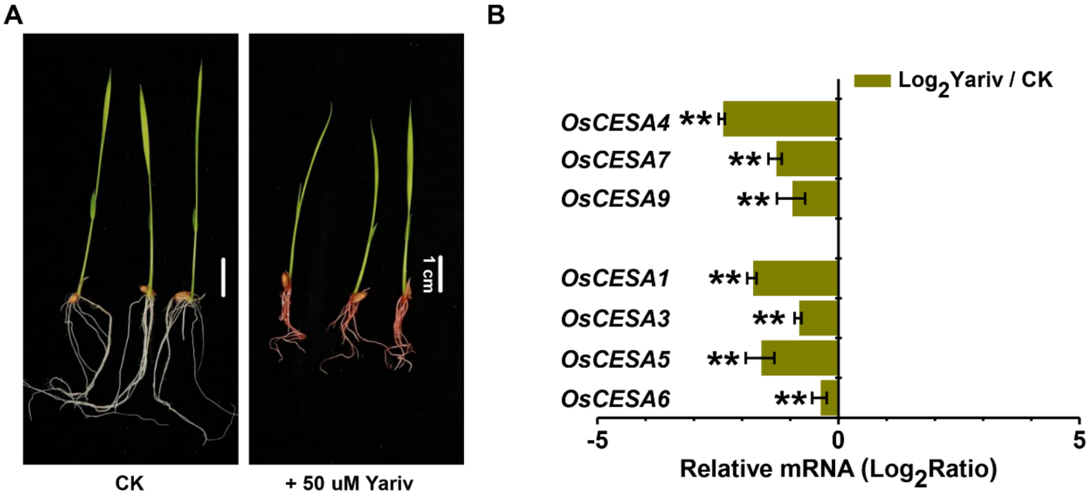

Supplement: eraa044_suppl_supplementary_tables_S1_S3_figures_S1_S8 [file eraa044_suppl_supplementary_tables_s1_s3_figures_s1_s8.pdf]
